# Surface Roughening Behavior of 6063 Aluminum Alloy during Bulging by Spun Tubes

**DOI:** 10.3390/ma10030299

**Published:** 2017-03-16

**Authors:** Yang Cai, Xiaosong Wang, Shijian Yuan

**Affiliations:** 1 School of Materials Science and Engineering, Harbin Institute of Technology, Harbin 150090, China; 108caiyang@163.com; 2 National Key Laboratory of Precision Hot Processing of Metals, Harbin Institute of Technology, Harbin 150001, China; syuan@hit.edu.cn

**Keywords:** surface roughening, grain size, strain, aluminum alloy tube, bulging

## Abstract

Severe surface roughening during the hydroforming of aluminum alloy parts can produce surface defects that severely restrict their application in the automobile and aerospace industry. To understand the relation between strain, grain size and surface roughness under biaxial stress conditions, hydro-bulging tests of aluminum alloy tubes were carried out, and the tubes with different grain sizes were prepared by a spinning and annealing process. The surface roughness was measured by a laser scanning confocal microscope to evaluate the surface roughening macroscopical behavior, and the corresponding microstructures were observed using electron back-scattered diffraction (EBSD) to reveal the roughening microscopic behavior. The results obtained show that the surface roughness increased with both strain and grain size under biaxial stress. No surface defects were observed on the surface when the grain size was less than 105 μm if the strain was less than 18%, or when the grain size was between 130 and 175 μm if the strain was less than 15.88% and 7.15%, respectively. The surface roughening microscopic behavior was identified as an inhomogeneous grain size distribution, which became more pronounced with increasing grain size and resulted in greater local deformation. Concentrated grain orientation also results in severe inhomogeneous deformation during plastics deformation, and serious surface roughening.

## 1. Introduction

Aluminum alloy has played an important role in lightening automobiles [1,2,3], with hydroformed aluminum tubes making it possible to create lightweight parts with spatial structures and complex sections [2,4,5,6] that can replace steel in high-end cars, and achieve energy savings and emission reductions. The first aluminum alloy rear axle subframe made by hydroforming was used in the 5-series BMW(Bayerische Motoren Werke), with its total weight of only 11.5 kg achieving a significant weight reduction when compared to steel [7]. Audi have since manufactured roof rails by hydroforming 6014 aluminum alloy and used these in their A2 and A8 series vehicles to reduce their body weight by 40% [8].

Surface roughening occurs on the surface of aluminum alloy tubes during hydroforming deformation, and when the surface roughening is severe enough, it produces surface defects such as “orange peel” or ridging and roping that affects the industrial production negatively [9,10,11]. It is widely accepted that such materials have an anisotropic behavior and different crystal orientations, which leads to incompatibilities of deformation arising from interactions between neighboring grains [12]. The surface roughening behavior attributable to the deformation of individual grains can generally be observed on a grain scale [13,14], and so the most basic influential factors include internal factors (mainly grain size) and external conditions (mainly strain) [15].

The factors that influence surface roughening have mostly been investigated through tensile deformation, bending experiments and also cup drawing tests [16,17,18,19,20,21]. Under a uniaxial stress state, the surface roughness first increases then decreases slightly with increasing strain, but increases linearly with increasing grain size [15,19]. Other studies have found that the surface roughness increases linearly with both increasing grain size and strain [17], but very few reports have paid attention to surface roughening under biaxial stress conditions. One study investigated the relationship between surface roughness and strain using numerical simulation, but this was neither verified by relevant experiments, nor did it consider the relationship between microstructure and surface roughness of tube during hydroforming [22].

In this study, bulging tests were carried out using 6063 aluminum alloy tubes with different microstructures to determine the quantitative relation between surface roughness, strain and grain size under biaxial stress. For this, tubes with different grain sizes and textures were prepared by spinning and annealing, and different strains were obtained by changing the area of the bulged tube. Measurements of surface roughness and observations of surface topography were also carried out using a confocal scanning laser microscope. Microstructural information was obtained using electron back-scattered diffraction (EBSD).

## 2. Experimental Procedure

### 2.1. Materials

**Figure 1:**
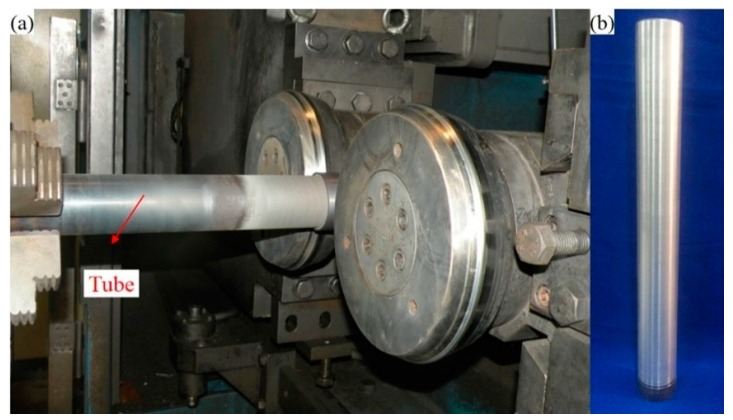
(**a**) Double-wheel spinning installation and (**b**) as-spun tube.

The grain size and texture of aluminum alloy can be changed through thermomechanical treatment, and so aluminum alloy tubes with different microstructures were obtained using the same spinning deformation, but with different heat treatments. The starting material for this was extruded 6063-T4 aluminum alloy tubes with a nominal outside diameter of 78 mm and nominal thickness of 3.5 mm, which were spun at room temperature with a nominal reduction of 5%–10% per pass to give a total reduction of 42.85% after six passes. The equipment used for this was the high-precision, double-wheeled spinning installation shown in Figure 1a, which had a spinning roller feed rate of 0.8 mm rev-1 and a mandrel speed of 250 rpm. The as-spun tubes (Figure 1b) were annealed at 350, 400 and 450 °C for 1 h and furnace-cooled to obtain tubes with three different microstructures, giving a total of four microstructures when combined with the initial tube. The alloy was conducted with T4 heat treatment before testing. The 6063 aluminum alloy used in the present investigation with chemical composition of 0.45% Si, 0.71% Mg, 0.03% Mn, 0.19% Fe, 0.03% Cu (wt %) and the balance Al.

### 2.2. Bulging

**Figure 2:**
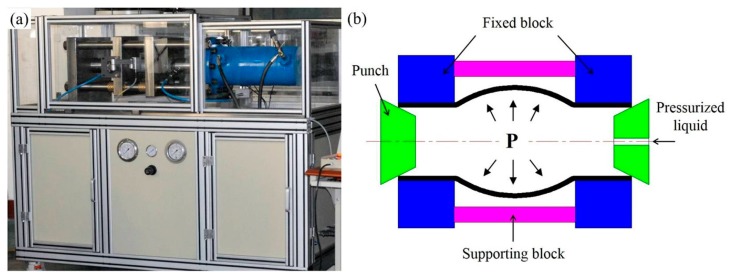
(**a**) Test unit and (**b**) schematic diagram for the hydro-bulging testing of tubes.

To investigate the surface roughening behavior of 6063 aluminum alloy tube during hydroforming, bulging experiments were carried out using the hydroformability test setup shown in Figure 2a. For this, tubes with a length–diameter ratio of 1.5 (i.e., a tube length of 112.5 mm) were fixed to a specially designed block, and then bulged using high pressure liquid. A schematic diagram of this hydro-bulging test is provided in Figure 2b. Prior to testing, the surface of the tube was mechanically polished to allow for measurement of any change in its surface roughness and facilitate observation of its morphology. A stress–strain curve was obtained from the bulge height and fluid pressure according to the method proposed by He et al. [23,24].

### 2.3. Measurement Method

To define a quantitative relation between the surface roughness and strain of aluminum alloy tubes with different grain sizes during hydroforming, their surfaces were divided randomly into 24 areas along the axial direction. The strain along this axial direction was then calculated during deformation from the reduction in thickness and circular expansion according to the law of constant volume. The arithmetical mean deviation of the profile (*R_a_*) is a reasonable representation of the roughening behavior of the material that is frequently used in industrial applications [25]. The surface roughness was therefore characterized in this study using the *R_a_* value obtained through direct measurement of a 2560 × 2560 μm target area using a laser scanning confocal microscope (OSL3000, which manufactured in Japanese Olympus, Tokyo, Japan) with a 5× objective lens, which gave a vertical resolution of 2 μm. The corresponding 2D and 3D surface topographies were also obtained.

Areas with an effective strain of approximately 7% were selected and information regarding their grain size, grain orientation and number of grain boundaries was acquired by EBSD using a Quanta 200 FEG operated at 20 KV with a 5.5 μm step size. Specimens for EBSD analysis were prepared by mechanical grinding and electropolishing in an electrolyte containing 20% perchloric acid in alcohol at a temperature of about −20 °C and a voltage of 25 V for 50 s.

## 3. Results and Discussion

### 3.1. Tube Obtained by Spinning and Heat Treatment

**Figure 3:**
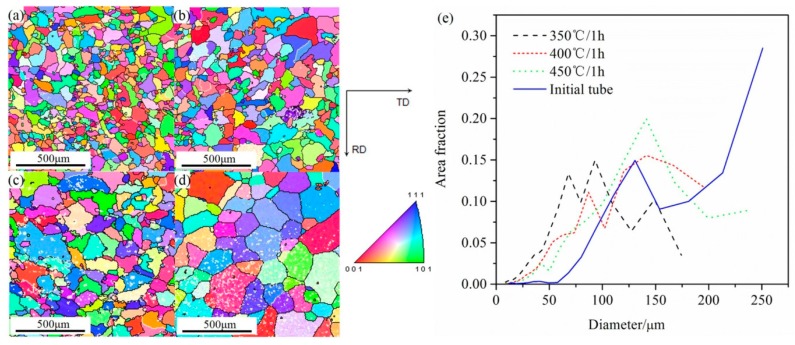
EBSD orientation maps of spun tube after annealing at (**a**) 350; (**b**) 400 and (**c**) 450 °C for 1 h; and (**d**) that of the initial tube; (**e**) Grain size distribution of each tube.

**Figure 4:**
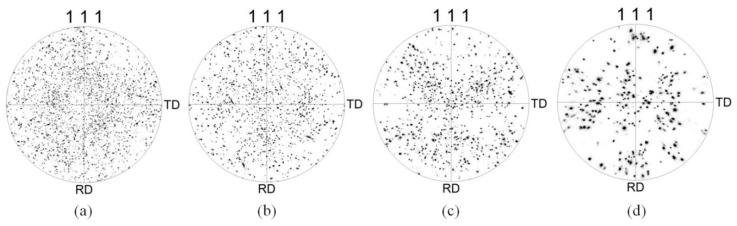
Grain orientation distributions of spun tube after annealing at (**a**) 350; (**b**) 400 and (**c**) 450 °C for 1 h; and (**d**) that of the initial tube.

The recrystallized microstructure created in the spun tube by annealing, and the grain size distribution of the initial tube and tubes annealed at different temperatures, were analyzed using the EBSD images shown in Figure 3. The analysis results listed in Table 1 show that the surface of the spun tube annealed at 350 °C had the smallest mean grain size of 80 μm, with a standard deviation (SD) of 28 μm. Increasing the annealing temperature to 400 °C resulted in a mean grain size of 105 μm (SD: 39 μm), whereas annealing at 450 °C produced a mean grain size of 130 μm (SD: 47 μm). For reference, the surface of the initial tube had a mean grain size of 175 μm (SD: 70 μm). The grain size distribution of each tube type is shown in Figure 3e. The corresponding grain orientation distributions of tubes before and after different annealing are shown in Figure 4. Figure 4a shows the grain orientation distribution of the spun tube annealed at 350 °C and the orientation distribution was very dispersive. Figure 4b is the result after increasing the annealing temperature to 400 °C, where the dispersion degree of grain orientation distribution was weakened. When the annealing temperature increased to 450 °C, the grain orientation distribution became relatively intensive. Figure 4d shows the grain orientation distribution of the initial tube where the orientation distribution was concentrated.

### 3.2. Roughening Macroscopical Behavior

**Figure 5:**
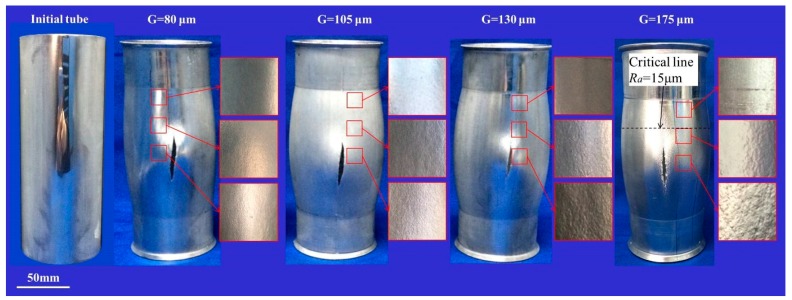
Bulged tubes and their respective surface roughening.

**Figure 6:**
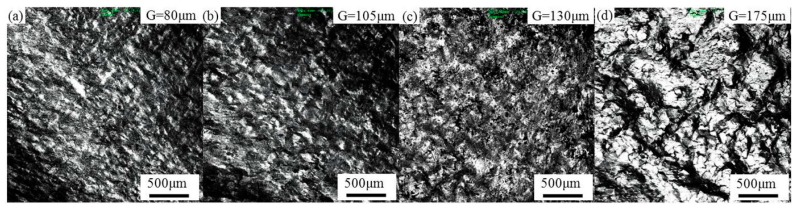
Surface morphology of bulged tubes with a mean grain size of (**a**) 80; (**b**) 105; (**c**) 130 and (**d**) 175 μm.

The surface roughness created on the mirror-finished surface of the tubes during bulging is shown in Figure 5, and their respective surface morphologies are shown in Figure 6. The “G” is the abbreviation of grain size in the whole paper. It is evident from this that a bigger grain size produces a more serious degree of surface roughening, with more pronounced concave–convex characteristics. The surface roughening of the initial tube with a grain size of 175 μm was the most serious, with only a small part of its surface at each end not being subject to “orange peel” defects. This same “orange peel” defect appeared near the fractures in the bulged tubes with grain sizes of 130 μm, but was larger in area in the former case. The tube with grain sizes of 80 and 105 μm experienced the least amount of surface roughening, with no evidence of “orange peel” defects anywhere on its surface. This suggests that there is a critical grain size for aluminum alloy tubes, below which “orange peel” defects will not occur. In the case of 6063 aluminum alloy tube, it would seem that keeping the mean grain size below 105 μm can help ensure that surface quality is controlled.

**Figure 7:**
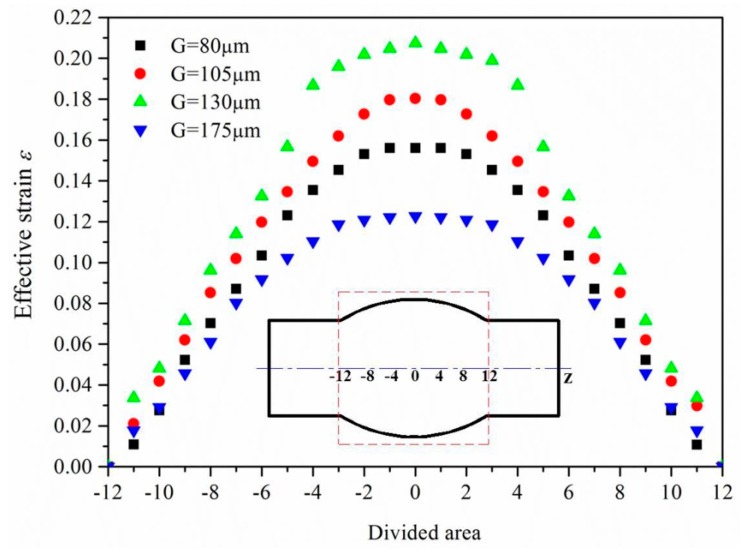
Effective strain distribution of bulged tubes with different grain sizes.

**Figure 8:**
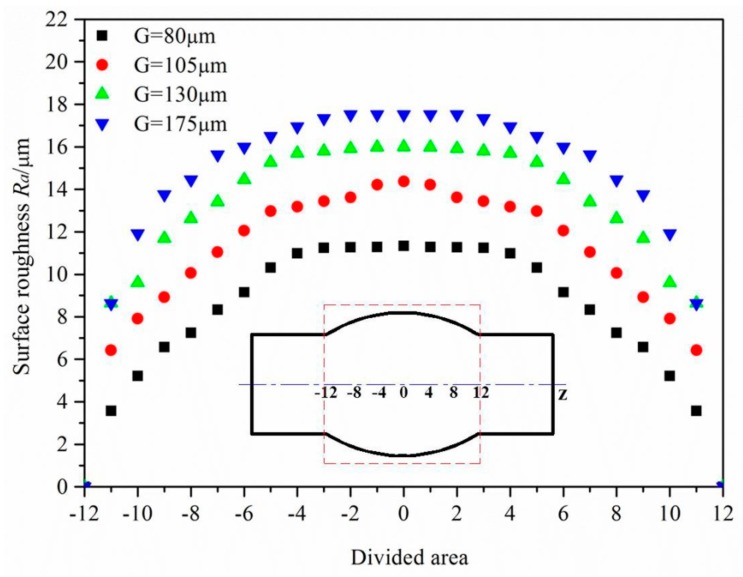
Surface roughness distribution of bulged tubes with different grain sizes.

The surfaces of the hydroformed tubes in Figure 5 also reveal that a greater degree of surface roughening occurs with a bigger strain, and so a critical strain exists in the grain size range of 130–175 μm at which “orange peel” defects first appear on the surface. By measuring the effective strain and surface roughness, the effective strain distribution and surface roughness distribution were determined, as shown in Figure 7 and Figure 8. It can be seen from this that the strain is less at the ends of the tube but more in the middle, creating a strain distribution that is symmetrical and continuous. As the surface roughness is really only influenced by strain in aluminum alloy tubes with a certain grain size, its distribution is similarly symmetrical and continuous.

**Figure 9:**
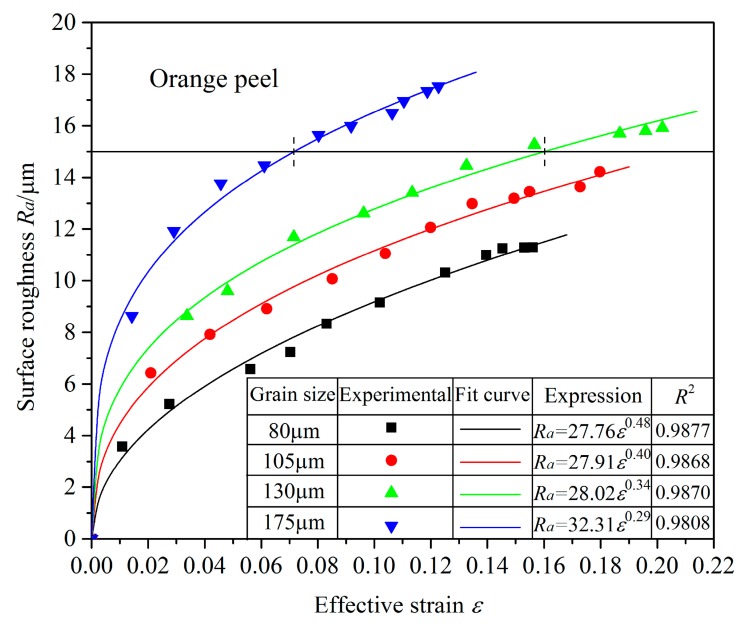
Relationship between surface roughness and effective strain of bulged tubes with different grain sizes.

**Figure 10:**
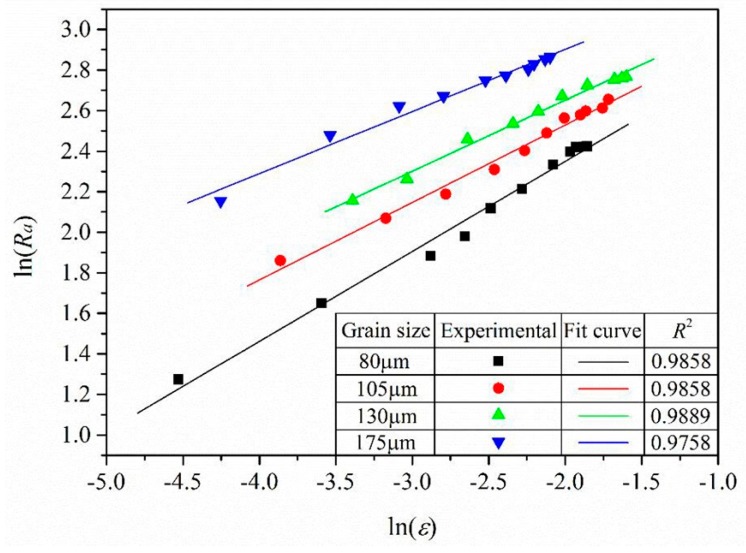
Relationship between ln(*R_a_*) and ln(*ε*) of bulged tubes with different grain sizes.

As the surface roughness corresponds to the effective strain of every area, the surface roughness–effective strain relationship of the bulged tubes was obtained. As shown in Figure 9, the surface roughness *R_a_* increased with effective strain *ε*, and their respective logarithms ln(*R_a_*) and ln(*ε*) have a linear relationship (Figure 10). This suggests that:

(1) $$R_{a}=f\left(G\right)\varepsilon^{g\left(G\right)}$$

**Figure 11:**
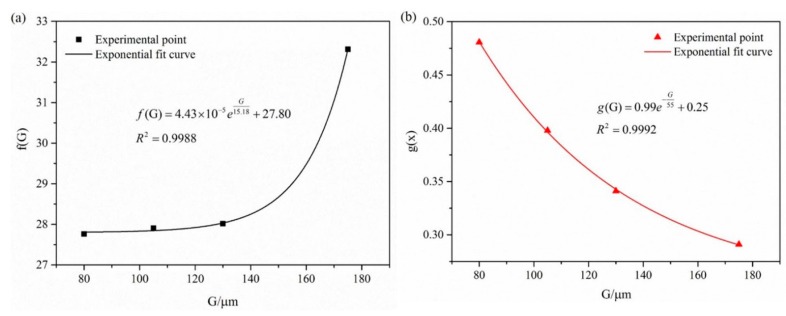
Fitting curves for (**a**) *f*(G) and (**b**) *g*(G).

The functions *f*(G) and *g*(G) are only related to grain size *G*, and the correspondence between *f*(G), *g*(G) and grain size *G* is shown in Figure 11. Using this, an expression for *f*(G) was obtained as:

(2) $$f\left(G\right)=4.43\times 10^{-5}e^{\frac{G}{15.18}}+27.80$$

where

(3) $$g(G)=0.99e^{-\frac{G}{55}}+0.25$$

Bringing Equations (2) and (3) into Equation (4), a relational expression for the surface roughness of 6063 aluminum alloy tube during bulging was obtained as:

(4) $$R_{a}=(4.43\times 10^{-5}e^{\frac{G}{15.18}}+27.80)\varepsilon^{0.99^{-\frac{G}{55}+0.25}}$$

**Figure 12:**
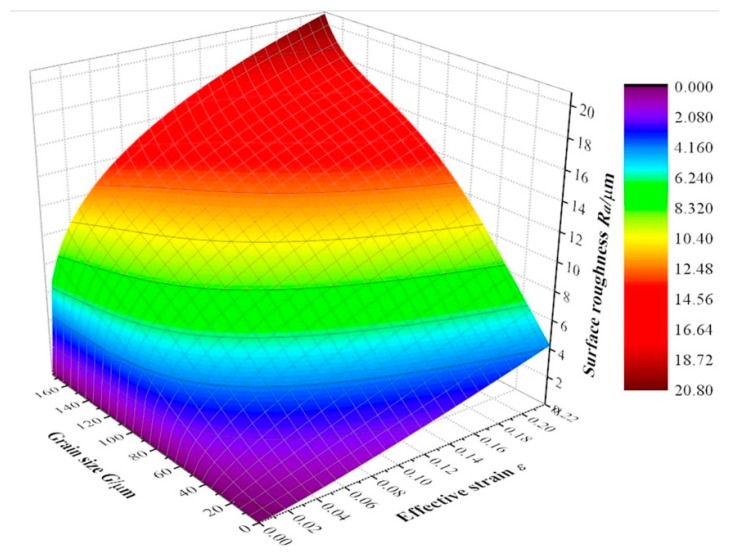
Curve surface showing the relation between surface roughness *R_a_*, grain size *G* and effective strain *ε*.

Here, *G* represents the grain size of the alloy (0 ≤ *G* ≤ 175 μm) and *ε* is the effective strain of the bulged tube (*ε* ≤ 0.22). Thus, the surface roughness during bulging clearly increases with both grain size and effective strain. The curve surface is shown in Figure 12.

From observations of the surface of the bulged tube with a grain size of 175 μm, it was found that surface roughening is not serious (15 μm) when the strain is less than 7.15%, but “orange peel” defects can occur when the strain is greater than this. Using a surface roughness of 15 μm as an index for the occurrence of “orange peel” defects, the critical strain for a grain size of 175 μm was found to be 7.15% according to Equation (4). Using this same approach, the critical strain for a grain size of 130 μm was found to be 15.88%. Thus, keeping the strain during the hydroforming of aluminum alloy structural components below these levels should ensure that “orange peel” defects are avoided.

### 3.3. Roughening Microscopic Behavior

**Figure 13:**
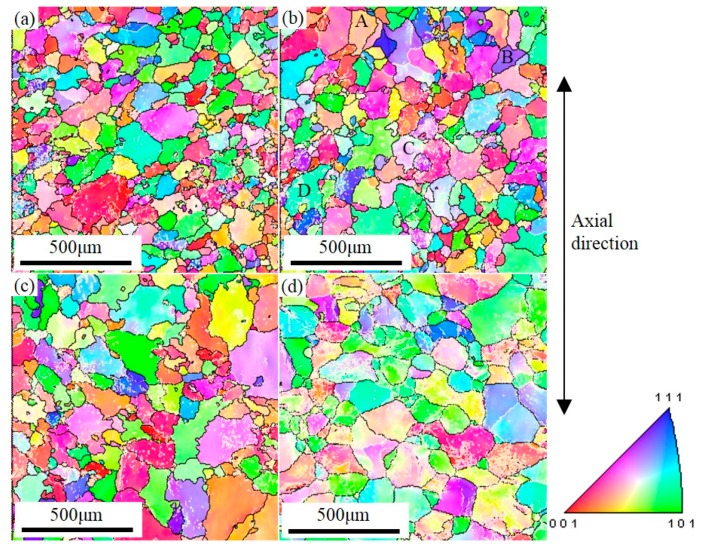
IPF maps of bulged tubes with a mean grain size and strain of (**a**) 80 μm and 7.52%; (**b**) 105 μm and 7.15%; (**c**) 130 μm and 7.03%; (**d**) 175 μm and 7.22%.

Using EBSD, an inverse pole figure (IPF) was calculated to describe the orientation and grain structure for an area at the surface of a bulged tube with a strain of ~7% and different grain sizes, as shown in Figure 13. Here, different colors represent different grain orientations, and so low-angle grain boundaries (LAGBs, 2°–15°) and high-angle grain boundaries (HAGBs, >15°) are represented by black and white lines, respectively.

Serious strain localization can be caused by the large standard deviation of grain size [26]. The standard deviation of grain size increases with the increasing average grain size as listed in Table 1, which suggests that the inhomogeneity of the grain size distribution is more serious if the average grain size is larger. Moreover, in the microstructure formed with a mean grain size of 80 μm shown in Figure 13a, there are more HAGBs involved in deformation. This, in turn, means that there are fewer LAGBs (38.4%) in the grains [27], and so the deformation is uniformly distributed in each grain. With an increase in mean grain size, the LAGBs increase and the deformation in each grain becomes relatively nonuniform, resulting in obvious local deformation. The local deformation in the microstructure with a mean grain size of 175 μm shown in Figure 13d is the most obvious of this. The surface roughening microscopic behavior is therefore the greater inhomogeneity of grain size distribution that occurs with increasing grain size, which results in enhanced local deformation. Consequently, the surface roughening increases with grain size. In addition, the grain orientation distribution also affects the surface roughening behavior of aluminum alloy during plastic deformation. Figure 4a shows that the grain orientation distribution was the most dispersive, and the surface roughening of the tube after bulging was the most slight. Figure 4d shows that the grain orientation distribution was the most concentrated, and the surface roughening was the most serious. Concentrated grain orientation results in severe inhomogeneous deformation during plastics deformation, and serious surface roughening.

## 4. Conclusions

The surface roughening behavior during tube bulging was investigated and the following conclusions were reached:

1. Under biaxial stress conditions, the surface roughness increases with increasing grain size. When the grain size is less than 105 μm, there is no “orange peel” on the surface, nor is there any when the grain size is 130 μm provided the strain is less than 15.88%. Similarly, when the grain size is 175 μm, there is no “orange peel” on the surface when the strain is less than 7.15%.
2. Under biaxial stress conditions, the surface roughness increases exponentially with strain. The relational expression for the surface roughness of 6063 aluminum alloy tube was determined to be $R_{a}=(4.43\times 10^{-5}e^{\frac{G}{15.18}}+27.80)\varepsilon^{0.99^{-\frac{G}{55}+0.25}}$. The surface roughness of hydroformed tubes can therefore be controlled by adjusting the initial grain size or corresponding strain, thereby improving the surface quality of the final structural component.
3. The surface roughening microscopic behavior is an increase in the inhomogeneity of the grain size distribution with increasing grain size, resulting in greater local deformation. Concentrated grain orientation also caused the serious surface roughening during plastics deformation. Thus, surface roughening increases with grain size, its deviation and grain orientation.

## Figures and Tables

**Table 1:** Grain size analysis of tubes prepared with different grain sizes.

Annealing Temperature	Mean Grain Size	Standard Deviation
350 °C	80 μm	28 μm
400 °C	105 μm	39 μm
450 °C	130 μm	47 μm
Initial tube	175 μm	70 μm
